# Evaluation of the effects of an offer of an incentive on the rate of questionnaire return: a nested RCT

**DOI:** 10.1186/1745-6215-16-S2-P97

**Published:** 2015-11-16

**Authors:** Pollyanna Hardy, Peter Brocklehurst

**Affiliations:** 1 National Perinatal Epidemiology Unit CTU, University of Oxford, Oxford, UK; 2 Institute for Women's Health, University College London, London, UK

## Introduction

A recent systematic review on the use of incentives to promote questionnaire return in clinical trials suggest they are effective [1], but not all studies have sufficient funds to use them. Promising an incentive once data are returned can reduce the cost burden of this approach, with possible further cost-savings if the offer were restricted to reminder letters only.

This study aims to evaluate the effect of promising a monetary incentive at first mail out versus a promise on reminder letters only.

## Methods

This is a randomised Study Within A Trial (SWAT) nested within BUMPES, a multicentre randomised controlled trial in women admitted to a labour ward, ≥37 weeks’ gestation and with a low dose epidural in situ. The follow-up postal questionnaire asked for information on the woman's health, wellbeing and health service use one year following the birth of their baby. Women who consented to be contacted were randomised to a promise of a monetary incentive at first mail out versus a promise on reminder letters only. Women were also given an option of completing the questionnaire online. The incentive was posted out on receipt of a completed questionnaire.

## Results

A total of 1,029 women were randomised into the SWAT. Final results, including the comparison of the proportion of questionnaires returned between randomised groups, will be presented at the conference.
